# Supplementary material for: H2O and CO2 Sorption in Ion-Exchange Sorbents: Distinct Interactions in Amine Versus Quaternary Ammonium Materials
Source: ACS Appl Mater Interfaces. 2025 Sep 9;17(38):53547–62. doi: 10.1021/acsami.5c12939 (PMC12466856; doi:10.1021/acsami.5c12939)
Supplement: Supplementary file 1 [file am5c12939_si_001.pdf]

Supporting Information

H_2O and CO_2 sorption in ion exchange sorbents: distinct interactions in amine versus quaternary ammonium materials

Golnaz Najaf Tomaraei[1], Sierra Binney [1], Ryan Stratton [1], Houlong Zhuang[2], and Jennifer L. Wade[1*]

[1] Department of Mechanical Engineering, Northern Arizona University, Flagstaff, Arizona, 86011, USA

[2] School for Engineering of Matter, Transport and Energy, Arizona State University, Tempe, Arizona 85287, USA

Table of Contents

Uncertainty Analysis 2

Supporting Figures and Tables 7

Figure S1. Representative raw data from simultaneous measurements. 8

Figure S2. Effect of PCO_2 (0, 400, and 3000 ppm) on water desorption in IRA900-RA at 25 °C. 9

Table S1. Surface area, average pore width, and total pore volume of Lewatit, IRA900-Cl, and IRA900-RA sorbents 9

Table S2. Water sorption and calorimetric enthalpy data for Lewatit. 9

Table S3. Water sorption and calorimetric enthalpy data for IRA900-RA. 10

Table S4. Water uptake and release comparison between IRA900-Cl and IRA900-RA at 25 °C. 10

Table S5. GAB model parameters obtained from fitting the experimental water sorption isotherm data for Lewatit. 10

Table S6. GAB model parameters obtained from fitting the experimental water desorption isotherm data for IRA900-RA. 10

Table S7. Water sorption and calorimetric enthalpy data for IRA900-Cl and IRA900-RA at 25°C. 11

Table S8. Water sorption and calorimetric enthalpy data for IRA900-RA at 25°C under 400 ppm and 3000 ppm CO_2 in N_2. 11

Table S9. Water sorption and desorption data for IRA900-RA at 25 °C under PCO_2 = 0, 400, and 3000 ppm in N_2. 11

Table S10. CO_2 uptake during water desorption and CO_2 release during water sorption in IRA900-RA at 25 °C under PCO_2 = 400 and 3000 ppm in N_2. 11

References 12

Uncertainty Analysis

This section provides a comprehensive analysis of uncertainty and error propagation associated with the key measured and derived quantities in this study, including mass, water uptake, enthalpy, and gas concentrations.[1] Sources of uncertainty include instrumental resolution, signal noise, calibration errors, and data processing techniques.

Uncertainty in mass measurement

The uncertainty in a mass measurement, U_{mass}, arises from two sources: instrumental resolution of the microbalance, $U_{res} = 0.001\, mg$, and the noise in baseline readings without any flow, estimated as the standard error of the mean over 9 observations:

$$U_{nse} = \frac{\sigma}{\sqrt{n}} = \frac{0.0015}{\sqrt{9}} = 0.0005\, mg$$

These two uncertainties are combined using the root-sum-square method:

$$U_{mass} = [U_{nse}^2 + U_{res}^2]^{0.5} = \sqrt{0.0005^2 + 0.001^2} \approx 0.001\, mg$$

Where the U_{res} comes from the resolution of the microbalance which is 0.001 mg.

Uncertainty in mass change, $\Delta m = m_2 - m_1$

Assuming equal uncertainty in the two mass measurements, the uncertainty in the mass change is:

$$U_{\Delta m} = \left[\left(\frac{\partial \Delta m}{\partial m_1}.U_{m_1}\right)^2 + \left(\frac{\partial \Delta m}{\partial m_2}.U_{m_2}\right)^2\right]^{0.5} = \left[U_{m_1}^2 + U_{m_2}^2\right]^{0.5} = \sqrt{2}\,U_{mass} \approx 0.002\, mg$$

Uncertainty in molar water uptake, n_{H_2O} $[mmol]$

In water sorption experiments, the molar water uptake, n_{H_2O} $[mmol]$, is calculated by dividing $\Delta m\,[mg]$ by the molecular weight of water (18.01 g/mol). Therefore, the uncertainty in n_{H_2O} is:

$$U_{n_{H_2O}} = \frac{1}{18.01}.U_{\Delta m} = 1.1 \times 10^{-4}\, mmol$$

Since water uptake is cumulative over multiple RH cycles, the uncertainty in n_{H_2O}, $U_{n_{H_2O}}$, grows as we move forward in cycles:

$$U_{n_{H_2O},k} = \sqrt{k}.\frac{1}{18.01}.U_{\Delta m} = \sqrt{k}.U_{n_{H_2O},1}$$

For instance, at the last cycle (12th cycle),

$$U_{n_{H_2O},12} = \sqrt{12}.U_{n_{H_2O},1} = 3.85 \times 10^{-4}\, mmol$$

Uncertainty in normalized water uptake, nq_{H_2O} $[mmol/g]$

Water uptake normalized to sample dry mass, M, is:

$$q_{H_2O} = \frac{1}{M}.n_{H_2O}\ [\frac{mmol}{g}]$$

Using error propagation:

$$U_{q_{H_2O},1} = \left[\left(\frac{\partial q_{H_2O}}{\partial M}.U_{mass}\right)^2 + \left(\frac{\partial q_{H_2O}}{\partial n_{H_2O}}.U_{n_{H_2O},1}\right)^2\right]^{0.5}$$

$$U_{q_{H_2O},1} = \left[\frac{n_{H_2O}^2}{M^4}.U_{mass}^2 + \frac{1}{M^2}.U_{n_{H_2O},1}{}^2\right]^{0.5}$$

For example, in the 1st cycle of water sorption experiment at 25 °C for IRA900-RA, we have:

M = 5.956 mg

$n_{H_2O,1} = -0.0337\ mmol$

$U_{mass} = 0.001\ mg$

$U_{n_{H_2O},1} = 1.1 \times 10^{-4}\ mmol$

Substituting the values, we get a representative value for $U_{q_{H_2O},1}$:

$$U_{q_{H_2O},1} = \left[\frac{0.0337^2}{5.956^4}.0.001^2 + \frac{1}{5.956^2}.(1.1 \times 10^{-4})^2\right]^{0.5} \approx 0.018\ mmol/g$$

At the 12th cycle:

$$U_{q_{H_2O},12} = \sqrt{12}.U_{q_{H_2O},1} = 0.064\ mmol/g$$

Uncertainty in Water Sorption Enthalpy per mass, Q_{st}

As described in Section 2, the raw heat flow signal is integrated over time to obtain Q_{raw} in units of µV.s. This is then converted to joules using the gallium sensitivity factor of the DSC (Ga-Sens):

$$Q_{con} = Q_{raw} \times Ga - Sens$$

The uncertainty in the converted heat, Q_{con}, is given by:

$$U_{Q_{con}} = \left[\left(\frac{\partial Q_{con}}{\partial Q_{raw}}.U_{Q_{raw}}\right)^2 + \left(\frac{\partial Q_{con}}{\partial Ga-Sens}.U_{Ga-Sens}\right)^2\right]^{0.5}$$

$$U_{Q_{con}} = \left[\left(Ga - sens\ .U_{Q_{raw}}\right)^2 + (Q_{raw}\ .U_{Ga-Sens})^2\right]^{0.5}$$

From a representative exothermic sorption signal from our experimental data we have: $Q_{raw} = -311.77\ \mu V \cdot s$ (the average of three integrations)

$U_{Q_{raw}} = 12.55\ \mu V \cdot s$ (the standard error of the mean from three integrations)

Ga-Sens = 0.001266 J/ μV·s

$U_{Ga-Sens} = 10^{-4}$ (the standard error of mean from three replicate Ga samples)

$$U_{Q_{con}} = [(0.001266 \times 12.55)^2 + (311.77 \times 10^{-4})^2]^{0.5} = 0.035\, J$$

Calorimetric water sorption enthalpy, Q_{st}, is calculated by dividing the converted heat by the corresponding mass change:

$$Q_{st} = \frac{Q_{con}}{\Delta m}$$

The associated uncertainty, $U_{Q_{st}}$, is given by:

$$U_{Q_{st}} = \left[\left(\frac{\partial Q_{st}}{\partial Q_{con}} . U_{Q_{con}}\right)^2 + \left(\frac{\partial Q_{st}}{\partial \Delta m} . U_{\Delta m}\right)^2\right]^{0.5}$$

$$U_{Q_{st}} = \left[\left(\frac{1}{\Delta m} . U_{Q_{con}}\right)^2 + \left(\frac{Q_{con}}{\Delta m^2} . U_{\Delta m}\right)^2\right]^{0.5}$$

From experimental data the average mass change associated with the above-mentioned exothermic sorption signal is:

$$\Delta m = 0.157\, mg$$

$$U_{\Delta m} = 0.002\, mg$$

$$Q_{con} = 0.001266 \times -311.77 = -0.395\, J$$

Substituting:

$$U_{Q_{st}} = \left[\left(\frac{1}{0.157} \times 0.035\right)^2 + \left(\frac{0.395}{0.157^2} \times 0.002\right)^2\right]^{0.5} \approx 0.225\, J/mg$$

The calculated water sorption enthalpy is:

$$Q_{st} = \frac{Q_{con}}{\Delta m} = \frac{0.001266 \times -311.77}{0.157} = -2.51\, J/mg$$

Therefore, a relative uncertainty of approximately 9% is used as a typical estimated for calorimetric water sorption enthalpy values in this work.

Uncertainty in gas flow rate and composition

The dry molar flow rate, $\dot{n}_{dry}$, is calculated from the total volumetric flow rate using the ideal gas law:

$$\dot{n}_{dry} = \frac{\dot{V}_{total} \times 10^{-6} \times P_{total}}{60 \times R \times T_{total}}$$

The uncertainty in $\dot{n}_{dry}$ due to uncertainty in volumetric flow rate $U_{\dot{V}_{total}} = 1\ sccm$ is:

$$U_{\dot{n}_{dry}} = \frac{10^{-6} \times P_{total}}{60 \times R \times T_{total}} \times U_{\dot{V}_{total}} = \frac{10^{-6} \times 101325}{60 \times 8.314 \times 273.15} \times 1 \approx 7.44 \times 10^{-7}\ mol/s$$

The actual input flow rate, $\dot{n}_{in}$, accounts for water vapor inclusion:

$$\dot{n}_{in} = \frac{\dot{n}_{dry}}{1 - H_2Obase \times 10^{-3}}$$

Where H_2O_{base} is the baseline water vapor composition in parts per thousand mole fraction. The uncertainty in $\dot{n}_{in}$ is propagated as:

$$U_{\dot{n}_{in}} = \left[\left(\frac{1}{1 - H_2Obase \times 10^{-3}} \times U_{\dot{n}_{dry}}\right)^2 + \left(\frac{\dot{n}_{dry} \times 10^{-3}}{(1 - H_2Obase \times 10^{-3})^2} \times U_{H_2Obase}\right)^2\right]^{0.5}$$

Where U_{H_2Obase} is estimated from the standard deviation over baseline segments with no water or CO_2 present. Typical values range from 2.4×10^{-5} to $3.3 \times 10^{-6}\ ppt$.

$$U_{H_2Obase} = \frac{\sigma_{H_2Obase}}{n}$$

The input CO_2 flow rate is:

$$\dot{n}_{CO_2-in} = CO_2base \times 10^{-6} \times \dot{n}_{in}$$

Where CO_2base is the baseline CO_2 mole fraction in parts per million.

$$U_{\dot{n}_{CO_2-in}} = \left[\left(10^{-6} \times \dot{n}_{in} \times U_{CO_2base}\right)^2 + \left(CO_2base \times 10^{-6} \times U_{\dot{n}_{in}}\right)^2\right]^{0.5}$$

U_{CO_2base} is based on the standard error over baseline segments with no water or CO_2 present. Typical values range from 2.3×10^{-5} to $7.7 \times 10^{-6}\ ppm$.

$$U_{CO_2base} = \frac{\sigma_{CO_2avg\ base}}{n}$$

The total output flow rate is:

$$\dot{n}_{out} = \frac{\dot{n}_{in}}{[1 - (H_2O - H_2Obase) \times 10^{-3} - (CO_2avg - CO_2base) \times 10^{-6}]}$$

The uncertainty in $\dot{n}_{out}$ is propagated as:

$$U_{\dot{n}_{out}} = \left[\left(\frac{1}{D} \times U_{\dot{n}_{in}}\right)^2 + \left(\frac{10^{-3} \times \dot{n}_{in}}{D^2} \times U_{H_2O}\right)^2 + \left(\frac{10^{-3} \times \dot{n}_{in}}{D^2} \times U_{H_2Obase}\right)^2 + \left(\frac{10^{-6} \times \dot{n}_{in}}{D^2} \times U_{CO_2avg}\right)^2 + \left(\frac{-10^{-6} \times \dot{n}_{in}}{D^2} \times U_{CO_2base}\right)^2\right]^{0.5}$$

Where $D = [1 - (H_2O - H_2Obase) \times 10^{-3} - (CO_2avg - CO_2base) \times 10^{-6}]$

The following uncertainties are used in the uncertainty for $U_{\dot{n}_{out}}$

$$U_{H_2O} = \left[{U_{res}}^2 + {U_{nse}}^2\right]^{0.5} = [0.001^2 + 0.008^2]^{0.5} = 0.008\ ppt$$

$$U_{CO_2avg} = \left[{U_{res}}^2 + {U_{nse}}^2 + {U_{smooth}}^2\right]^{0.5} = \left[0.01^2 + 0.05^2 + \left(\frac{0.05}{\sqrt{41}}\right)^2\right]^{0.5} = 0.05\ ppm$$

The uncertainties in H_2O and CO_2avg signals include two main contributions: the instrument resolution (U_{res}) and the signal noise (U_{nse}). U_{res} is 0.001 ppt for H_2O and 0.01 ppm for CO_2avg. U_{nse} accounts for fluctuations in sensors readings and is calculated as the standard error of the H_2O or CO_2avg signal over a baseline period under UHP N_2 flow. For CO_2avg, the signal is smoothened using a moving average (movmean in MATLAB) over 41 points, a smoothing-related uncertainty term $U_{smooth} = \frac{U_{nse}}{\sqrt{41}}$ is also added to the formula for U_{CO_2avg}.

The CO_2 output flow rate, $\dot{n}_{CO_2-out}$, and its uncertainty, $U_{\dot{n}_{CO_2-out}}$, are given by:

$$\dot{n}_{CO_2-out} = CO_2avg \times 10^{-6} \times \dot{n}_{out}$$

$$U_{\dot{n}_{CO_2-out}} = \left[\left(10^{-6} \times \dot{n}_{out} \times U_{CO_2avg}\right)^2 + \left(CO_2avg \times 10^{-6} \times U_{\dot{n}_{out}}\right)^2\right]^{0.5}$$

The CO_2 (de)sorption flow rate by the sample, $\dot{n}_{CO_2}$, and its uncertainty, $U_{\dot{n}_{CO_2}}$, are given by:

$$\dot{n}_{CO_2} = \dot{n}_{CO_2-out} - \dot{n}_{CO_2-in}$$

$$U_{\dot{n}_{CO_2}} = \left[\left(U_{\dot{n}_{CO_2-out}}\right)^2 + \left(U_{\dot{n}_{CO_2-in}}\right)^2\right]^{0.5}$$

The total amount of CO_2 (de)sorbed by the sample, n_{CO_2}, is calculated by integrating $\dot{n}_{CO_2}$ over time.

$$n_{CO_2} = \sum_i \left(time_i \times \dot{n}_{CO_{2}\,i} \right)$$

$$U_{n_{CO_2}} = \left[\sum_i \left(time_i \times U_{\dot{n}_{CO_{2}\,i}} \right)^2 \right]^{0.5}$$

Converting to mass, we have:

$$m_{CO_2} = 44 \times 10^3 \times n_{CO_2}$$

$$U_{m_{CO_2}} = 44 \times 10^3 \times U_{n_{CO_2}}$$

Water mass is determined by subtracting CO_2 mass from total mass measured by TGA.

$$m_{H_2O} = m_{TGA} - m_{CO_2}$$

$$U_{m_{H_2O}} = \left[\left(U_{m_{TGA}} \right)^2 + \left(U_{m_{CO_2}} \right)^2 \right]^{0.5}$$

$U_{m_{TGA}} = U_{\Delta m} = 0.002\ mg$, from previous analysis of uncertainty in mass change.

Supporting Figures and Tables

Figure S1 illustrates how mass, heat flow, RH, and evolved gas concentrations were simultaneously monitored and analyzed during temperature and RH controlled experiments. This representative raw data corresponds to the isotherm experiment on IRA900-RA at 25 °C in the presence of 400 ppm CO_2 in the background gas. Coupled H_2O desorption-CO_2 uptake as well as H_2O sorption-CO_2 release is evident in the gas concentration profiles, highlighting the material's moisture-swing responsiveness.

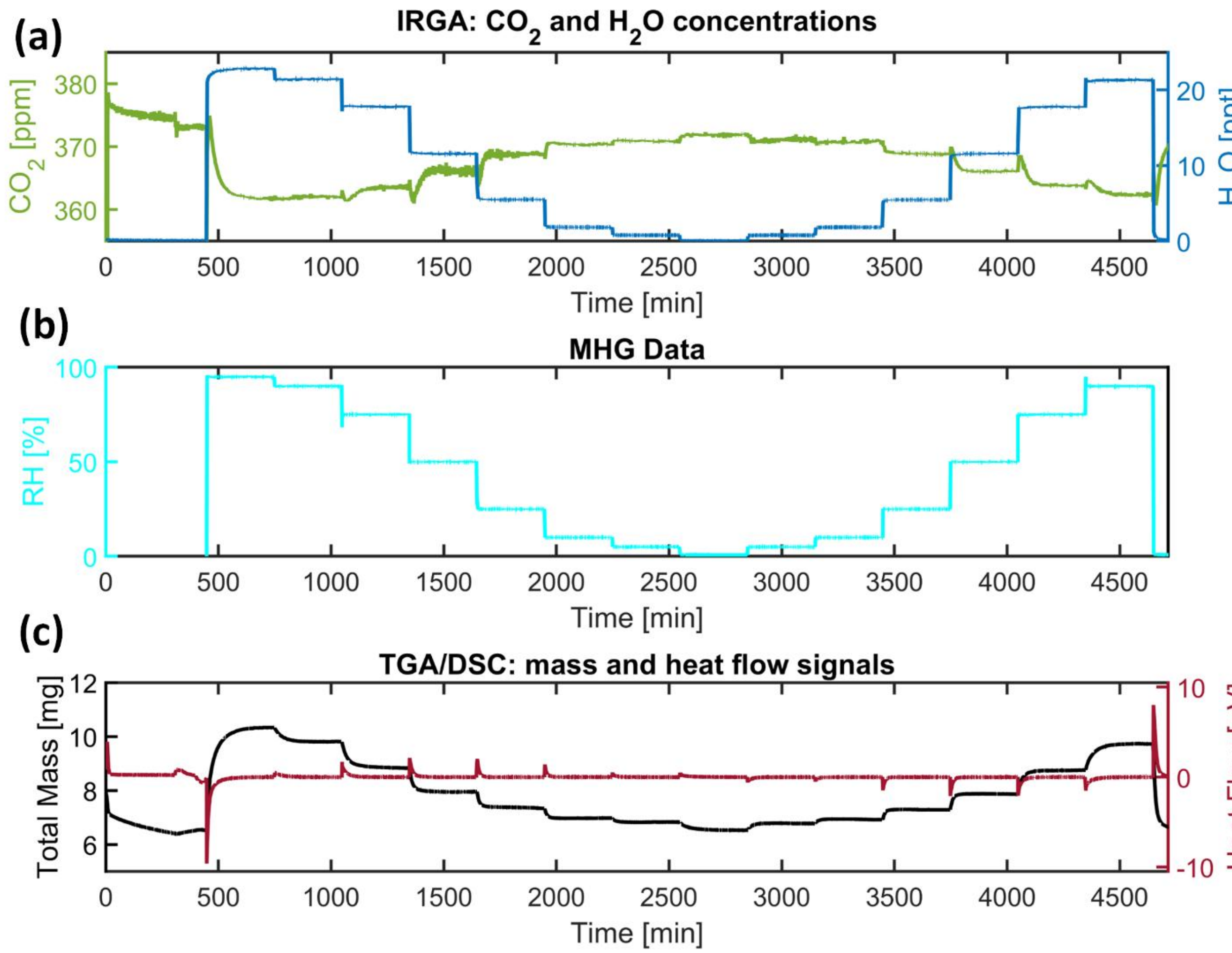

Figure S1. Representative raw data from simultaneous measurements. (a) IRGA real time concentration measurements showing CO_2 [ppm] (green, left axis) and H_2O [ppt] (blue, right axis). (b) Relative humidity [%] profile recorded by the MHG. (c) TGA data showing changes in total mass [mg] (black, left axis) and corresponding heat flow signal [μV] (red, right axis) during the water sorption isotherm experiments.

Figure S2 complements Figure 12 in the main text by showing that drying-induced water desorption is coupled with CO_2 uptake during isotherm experiments under mixed N_2/CO_2 gas conditions.

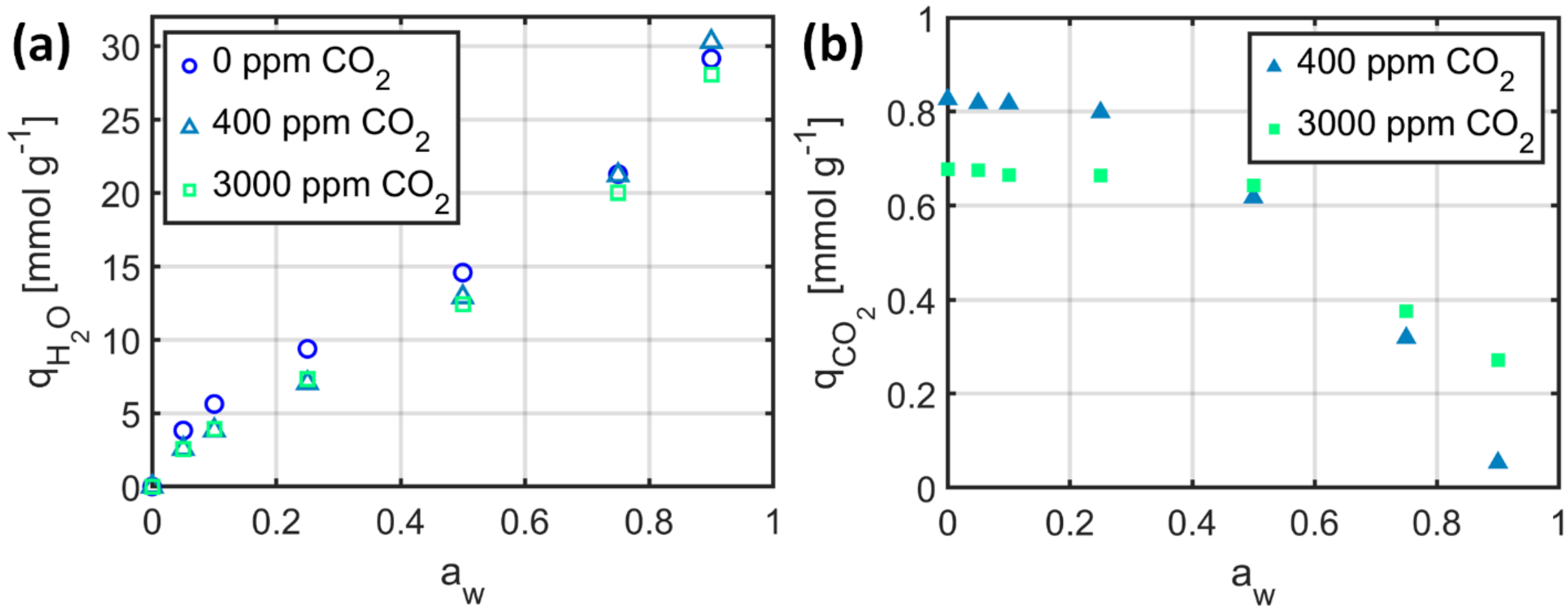

Figure S2. Effect of PCO_2 (0, 400, and 3000 ppm) on water desorption in IRA900-RA at 25 °C. (a) Water desorption isotherms during dehydration (RH 95% to 0%). (b) CO_2 uptake during water desorption under PCO_2 = 400 and 3000 ppm in N_2.

Table S1. Surface area, average pore width, and total pore volume of Lewatit, IRA900-Cl, and IRA900-RA sorbents derived from N_2 adsorption-desorption isotherms using BET and BJH analysis.

**Sample**	**BET surface area [m^2 g^{-1}]**	**Total pore volume [cm^3 g^{-1}]**	**Average pore width [nm]**
**Lewatit**	36.7	0.31	30.5
**IRA900-RA**	8.3	0.13	52.1
**IRA900-Cl**	11.5	0.15	45.4

Table S2. Water sorption and calorimetric enthalpy data for Lewatit. λ_{H_2O} (mmol H_2O per mmol of active site) and the corresponding integral heats of water sorption measured by direct calorimetry at different RH levels and temperatures.

RH [%]	12 °C				25 °C				40 °C			
	$\lambda_{H_2O}^{ads}$	$\lvert Q_{st}^{ads}\rvert$ [kJ/mol]	$\lambda_{H_2O}^{des}$	$\lvert Q_{st}^{des}\rvert$ [kJ/mol]	$\lambda_{H_2O}^{ads}$	$\lvert Q_{st}^{ads}\rvert$ [kJ/mol]	$\lambda_{H_2O}^{des}$	$\lvert Q_{st}^{des}\rvert$ [kJ/mol]	$\lambda_{H_2O}^{ads}$	$\lvert Q_{st}^{ads}\rvert$ [kJ/mol]	$\lambda_{H_2O}^{des}$	$\lvert Q_{st}^{des}\rvert$ [kJ/mol]
0			0.47	54.26			-0.16	47.81			0.55	62.31
5	0.30	55.94	0.90	56.85	0.22	55.56	0.21	49.02	0.14	54.88	0.79	57.57
10	0.59	54.75	1.16	53.59	0.32	76.47	0.53	48.17	0.43	58.19	1.05	47.15
25	1.06	53.53	1.84	59.71	0.82	46.19	1.23	41.54	0.86	43.35	1.65	43.19
50	1.98	54.42	2.87	49.56	1.64	46.81	2.19	48.89	1.68	41.15	2.56	39.54
75	2.96	47.88	3.65	45.04	2.67	41.07	2.92	38.98	2.85	45.41	NS[a]	
90	4.28	47.46			3.53	44.05			3.63			

[a]NS: Not stable. After exposure to 90% RH at 40 °C, the high water vapor pressure resulted in transient instability in TGA signal during the subsequent 75% RH desorption segment. The signal stability was recovered in later RH steps. The cumulative λ_{H_2O} values for subsequent RH levels reflect mass change from the last reliable segment (90% during sorption).

Table S3. Water sorption and calorimetric enthalpy data for IRA900-RA. λ_{H2O} (mmol H_2O per mmol of active site) and corresponding integral heats of water sorption measured by direct calorimetry at different RH levels and temperatures.

RH [%]	12 °C				25 °C				40 °C			
	$\lambda_{H_2O}^{ads}$	$\lvert Q_{st}^{ads}\rvert$ [kJ/mol]	$\lambda_{H_2O}^{des}$	$\lvert Q_{st}^{des}\rvert$ [kJ/mol]	$\lambda_{H_2O}^{ads}$	$\lvert Q_{st}^{ads}\rvert$ [kJ/mol]	$\lambda_{H_2O}^{des}$	$\lvert Q_{st}^{des}\rvert$ [kJ/mol]	$\lambda_{H_2O}^{ads}$	$\lvert Q_{st}^{ads}\rvert$ [kJ/mol]	$\lambda_{H_2O}^{des}$	$\lvert Q_{st}^{des}\rvert$ [kJ/mol]
0			0.00	56.33			0.00	48.36			0.00	55.09
5	0.79	52.49	0.83	61.95	0.78	59.39	1.11	50.99	0.79	65.07	1.13	55.11
10	1.18	55.21	1.30	56.56	1.29	50.81	1.62	47.68	1.23	57.59	1.64	51.84
25	2.11	50.88	2.35	44.23	2.39	47.97	2.72	44.11	2.13	54.69	2.66	51.32
50	3.82	56.01	3.97	50.48	3.86	45.06	4.22	44.69	3.45	49.59	4.04	47.38
75	5.92	40.22	6.14	43.61	5.69	47.05	6.14	40.11	5.42	45.30	5.91	42.97
90	7.79	44.57	8.01		7.93	41.26	8.37		5.97	45.65	6.51	

Table S4. Water uptake and release comparison between IRA900-Cl and IRA900-RA at 25 °C. %Δ values are relative to IRA900-RA. To obtain normalized hysteresis, the absolute differences between cumulative water uptake and release during sorption and desorption at each RH level are calculated and normalized by desorption values.

RH [%]	$q_{H_2O}^{ads}$ [mmol g^{-1}]			$q_{H_2O}^{des}$ [mmol g^{-1}]			Normalized hysteresis [%]	
	IRA900-Cl	IRA900-RA	%Δ_ads	IRA900-Cl	IRA900-RA	%Δ_des	IRA900-Cl	IRA900-RA
5	2.72	2.75	1.11	3.39	3.87	12.47	19.80	29.02
10	4.54	4.53	-0.28	5.01	5.66	11.52	9.42	20.08
25	8.10	8.36	3.09	8.37	9.51	12.01	3.17	12.09
50	13.06	13.52	3.38	12.94	14.76	12.34	0.96	8.40
75	19.65	19.90	1.26	19.50	21.48	9.24	0.81	7.34
90	27.00	27.76	2.74	26.92	29.31	8.16	0.31	5.28

Table S5. GAB model parameters obtained from fitting the experimental water sorption isotherm data for Lewatit.

**Temperature [°C]**	**q_m [mmol/g]**	**k**	**c**	**Normalized root mean square error (NRMSE)**
12	3.08	0.75	6.77	0.013
25	4.20	0.62	2.94	0.006
40	5.00	0.57	2.7	0.012

Table S6. GAB model parameters obtained from fitting the experimental water desorption isotherm data for IRA900-RA.

**Temperature [°C]**	**q_m [mmol/g]**	**k**	**c**	**Normalized root mean square error (NRMSE)**
12	11.34	0.7	7.80	0.007
25	10.36	0.73	13.81	0.005
40	9.74	0.74	15.72	0.007

Table S7. Water sorption and calorimetric enthalpy data for IRA900-Cl and IRA900-RA at 25°C. λ_{H_2O} and corresponding integral heats of water sorption measured by direct calorimetry at different RH levels.

RH [%]	IRA900-Cl				IRA900-RA			
	$\lambda_{H_2O}^{ads}$	$\lvert Q_{st}^{ads} \rvert$ [kJ/mol]	$\lambda_{H_2O}^{des}$	$\lvert Q_{st}^{des} \rvert$ [kJ/mol]	$\lambda_{H_2O}^{ads}$	$\lvert Q_{st}^{ads} \rvert$ [kJ/mol]	$\lambda_{H_2O}^{des}$	$\lvert Q_{st}^{des} \rvert$ [kJ/mol]
0			0.00	43.42			0.00	48.36
5	0.78	54.96	0.97	46.00	0.78	59.39	1.11	50.99
10	1.30	49.52	1.43	45.02	1.29	50.81	1.62	47.68
25	2.31	45.94	2.39	46.97	2.39	47.97	2.72	44.11
50	3.73	44.02	3.70	45.19	3.86	45.06	4.22	44.69
75	5.62	42.39	5.57	42.44	5.69	47.05	6.14	40.11
90	7.71	38.44	7.69		7.93	41.26	8.37	

Table S8. Water sorption and calorimetric enthalpy data for IRA900-RA at 25°C under 400 ppm and 3000 ppm CO_2 in N_2. λ_{H_2O} and corresponding integral heats of water sorption measured by direct calorimetry at different RH levels.

RH [%]	PCO_2 = 400 ppm				PCO_2 = 3000 ppm			
	$\lambda_{H_2O}^{ads}$	$\lvert Q_{st}^{ads} \rvert$ [kJ/mol]	$\lambda_{H_2O}^{des}$	$\lvert Q_{st}^{des} \rvert$ [kJ/mol]	$\lambda_{H_2O}^{ads}$	$\lvert Q_{st}^{ads} \rvert$ [kJ/mol]	$\lambda_{H_2O}^{des}$	$\lvert Q_{st}^{des} \rvert$ [kJ/mol]
0			0.00	47.14			0.00	58.15
5	0.61	53.59	0.74	61.10	0.66	61.68	0.72	51.50
10	0.95	50.83	1.09	45.99	1.16	45.68	1.12	48.87
25	1.92	45.25	2.01	41.26	2.15	47.38	2.09	44.62
50	3.46	44.52	3.68	42.15	3.65	43.22	3.55	40.89
75	5.91	42.31	6.06	37.71	5.91	42.33	5.72	45.25
90	8.45	37.49	8.65		8.30	40.87	8.01	

Table S9. Water sorption and desorption data for IRA900-RA at 25 °C under PCO_2 = 0, 400, and 3000 ppm in N_2.

RH [%]	$q_{H_2O}^{des}$ [mmol g^{-1}] @ 25 ℃			$q_{H_2O}^{ads}$ [mmol g^{-1}] @ 25 ℃		
	PCO_2 [ppm]			PCO_2 [ppm]		
	0	400	3000	0	400	3000
5	3.82	2.57	2.54	2.67	2.14	2.32
10	5.62	3.83	3.92	4.14	3.32	4.07
25	9.38	7.04	7.30	7.96	6.72	7.52
50	14.56	12.90	12.41	12.55	12.10	12.77
75	21.27	21.20	20.03	18.78	20.67	20.70
90	29.14	30.28	28.05	26.39	29.57	29.04

Table S10. CO_2 uptake during water desorption and CO_2 release during water sorption in IRA900-RA at 25 °C under PCO_2 = 400 and 3000 ppm in N_2.

RH [%]		$q_{CO_2}^{ads}$ [mmol g^{-1}] during H_2O desorption		$q_{CO_2}^{des}$ [mmol g^{-1}] during H_2O sorption
	PCO_2 = 400 ppm	PCO_2 = 3000 ppm	PCO_2 = 400 ppm	PCO_2 = 3000 ppm
0		0.83	0.68	

5	0.82	0.68	0.82	0.68
10	0.82	0.67	0.82	0.60
25	0.80	0.66	0.75	0.59
50	0.62	0.64	0.60	0.49
75	0.32	0.38	0.26	0.18
90	0.05	0.27	0.03	0.00

References

(1) Figliola, R. S.; Beasley, D. E. *Theory and Design for Mechanical Measurements*, Seventh edition.; Wiley: New York, 2020.
